# Anammox with alternative electron acceptors: perspectives for nitrogen removal from wastewaters

**DOI:** 10.1007/s10532-023-10044-3

**Published:** 2023-07-12

**Authors:** Sergio J. Ponce-Jahen, Bibiana Cercado, Edson Baltazar Estrada-Arriaga, J. Rene Rangel-Mendez, Francisco J. Cervantes

**Affiliations:** 1 Laboratory for Research on Advanced Processes for Water Treatment, Engineering Institute, Campus Juriquilla, Universidad Nacional Autónoma de México (UNAM), Blvd. Juriquilla 3001, 76230 Querétaro, Mexico; 2 Centro de Investigación y Desarrollo Tecnológico en Electroquímica S.C., Parque Tecnológico Querétaro Sanfandila, Querétaro 76703 Pedro Escobedo, Mexico; 3 Subcoordinación de Tratamiento de Aguas Residuales, Instituto Mexicano de Tecnología del Agua, Paseo Cuauhnáhuac 8532, Progreso, C.P. 62550 Morelos, Mexico; 4 División de Ciencias Ambientales, Instituto Potosino de Investigación Científica y Tecnológica (IPICyT), Camino a la Presa San José 2055, Col. Lomas 4ª Sección, SLP78216 San Luis Potosí, Mexico

**Keywords:** Ammonium removal, Anammox, Bioelectrochemical systems, Extracellular electron transfer, Wastewater treatment.

## Abstract

In the context of the anaerobic ammonium oxidation process (anammox), great scientific advances have been made over the past two decades, making anammox a consolidated technology widely used worldwide for nitrogen removal from wastewaters. This review provides a detailed and comprehensive description of the anammox process, the microorganisms involved and their metabolism. In addition, recent research on the application of the anammox process with alternative electron acceptors is described, highlighting the biochemical reactions involved, its advantages and potential applications for specific wastewaters. An updated description is also given of studies reporting the ability of microorganisms to couple the anammox process to extracellular electron transfer to insoluble electron acceptors; particularly iron, carbon-based materials and electrodes in bioelectrochemical systems (BES). The latter, also referred to as anodic anammox, is a promising strategy to combine the ammonium removal from wastewater with bioelectricity production, which is discussed here in terms of its efficiency, economic feasibility, and energetic aspects. Therefore, the information provided in this review is relevant for future applications.

## Introduction

Anammox is a microbial process driven by autotrophic bacteria, referred to as anammox bacteria, which remove nitrogen contained in wastewater in the form of ammonium (NH_4_^+^-N) and nitrite (NO_2_^−^-N) (Mulder 1995). In this process, nitrite serves as electron acceptor combined with ammonium to produce molecular nitrogen (the only environmentally friendly form of nitrogen), under anaerobic conditions, with the release of a small fraction of nitrate (NO_3_^−^-N) as a by-product (Qiao et al. 2009; Kartal et al. 2013). Regarding biomass production, it occurs through bicarbonate or carbon dioxide as the sole carbon source (Weralupitiya et al. 2021). The stoichiometry of the overall anammox reaction is represented by the following equation (Eq. 1) (Strous et al. 1998).This overall reaction is the net sum of two partial reactions, the ammonium oxidation coupled to nitrite reduction (Eq. 2) and bicarbonate fixation into cell mass (Eq. 3). Anammox′ is considered an economic, efficient, and environmentally friendly process (Yuan et al. 2021) because compared with the traditional nitrification-denitrification, anammox saves resources by requiring less aeration, no sources of organic carbon, and by producing less sludge than the traditional systems, which in turn lower operating costs and greenhouse gases emissions (Nsenga Kumwimba et al. 2020).

1 $${\text{NH}}_{{\text{4}}} ^{ + } + {\text{ 1}}.{\text{32NO}}_{{\text{2}}} ^{ - } + 0.0{\text{66HCO}}_{{\text{3}}} ^{ - } + {\text{ }}0.{\text{13H}}^{ + } \to {\text{1}}.0{\text{2N}}_{{\text{2}}} + {\text{ }}0.{\text{26NO}}_{{\text{3}}} ^{ - } + {\text{ }}0.0{\text{6CH}}_{{\text{2}}} {\text{O}}_{{0.{\text{5}}}} {\text{N}}_{{0.{\text{15}}}} + {\text{2}}.0{\text{3 H}}_{{\text{2}}} {\text{O}}~~~\Delta {\text{G}}^{{{\text{'0}}}} = - {\text{275 kJ}}/{\text{mol}}$$

2 $${\text{NH}}_{{\text{4}}} ^{ + } + {\text{ NO}}_{{\text{2}}} ^{ - } \to {\text{N}}_{{\text{2}}} + {\text{ 2H}}_{{\text{2}}} {\text{O}}~~~~~~~~\Delta {\text{G'}}^{{\text{0}}} = {\text{ }} - {\text{357 kJ}}/{\text{mol}}$$

3 $$0.{\text{27NO}}_{{\text{2}}} ^{ - } + {\text{ }}0.0{\text{66HCO}}_{{\text{3}}} ^{ - } \to 0.{\text{26NO}}_{{\text{3}}} ^{ - } + {\text{ }}0.0{\text{66CH}}_{{\text{2}}} {\text{O}}_{{0.{\text{5}}}} {\text{N}}_{{0.{\text{15}}~~~~~~~~~~~~~~}} \Delta {\text{G'}}^{0} = {\text{ }} + {\text{82 kJ}}/{\text{mol}}$$

## Anammox bacteria

Anammox bacteria are anaerobic, chemolithoautotrophic and spherical microorganisms that belong to the order Brocadiales within the phylum *Planctomycetes* (Jetten et al. 2009). It has been proven that the distribution of anammox bacteria is ubiquitous in environments as diverse as freshwater reserves, marine environments, terrestrial ecosystems, wastewater treatment systems and sediments (Strous et al. 1999; Schmid et al. 2001; Kuypers et al. 2003; Li et al. 2010; Brandsma et al. 2011; Speth et al. 2017).

So far, more than 25 anammox species have been discovered, which belong to six genera. Among them, five genera have been enriched from activated sludge and freshwater environments: Brocadia (Strous et al. 1999), Kuenenia (Schmid et al. 2000), Jettenia (Quan et al. 2008), Anammoxoglobus (Kartal et al. 2007) and Anammoximicrobium (Khramenkov et al. 2013). The sixth, Scalindua (Kuypers et al. 2003), is frequently detected in natural habitats, especially in marine sediments and oxygen minimum zones.

The distinctive red color of the anammox bacteria is due to the high cytochrome *c* proteins in their cells (Jetten et al. 2009). Because of the high number of cytochromes expressed by the bacteria, it has been proposed that these proteins participate in the transport of electrons among the various parts of the metabolic machinery (van Niftrik et al. 2008). In fact, it was reported that the cytochromes *c* found in anammox bacteria appear to be homologous to the multi-heme cytochromes of *Geobacter* and *Shewanella*, which are responsible for boosting the transfer of electrons with solid-phase electron acceptors (Ferousi et al. 2017).

## Additional microorganisms detected in anammox processes

Even though most investigations have postulated anammox bacteria as the main microorganisms responsible for performing the anammox process, they are extremely difficult to isolate. As a result, it is common that heterotrophs are also present in anammox systems. Molecular methods have shown that heterotrophic bacteria belonging to different phyla (*Acidobacteria*, *Bacteroidetes Chloroflexi*, *Chlorobi, Nitrospirae* and *Proteobacteria*) also comprise an important fraction of the community performing this process (Gonzalez-Martinez et al. 2015; Gonzalez-Gil et al. 2015; Lawson et al. 2017). Although these microorganisms have differences in nutrition and metabolic profiles, and they form a complex interaction network (Zhang et al. 2020), their role in anammox processes is not yet clear. However, some interactions have been proposed: (1) anammox bacteria and heterotrophic bacteria may support nitrogen removal from wastewater because heterotrophic community could contribute to the removal of debris and peptides produced by the anammox bacteria (Lawson et al. 2017), and (2) the heterotrophic microorganisms may encode the ability to breathe the nitrate produced during the synthesis of anammox biomass by partial denitrification (the reduction of nitrate back to nitrite), the produced nitrite can then be reduced by anammox (Speth et al. 2017). In contrast, the interactions can also be negative, like competition for substrates, which promotes changes in the abundance of anammox bacteria and decreases in nitrogen removal efficiency during the anammox process (Feng et al. 2019; Zhang et al. 2020).

## Anammox metabolism

### Catabolism

Anammox catabolism takes place within the anammoxosome (Niftrik et al. 2004). So far, two catabolic pathways have been described in anammox bacteria. In route 1, van de Graaf et al. (1997) proposed a three-step model with N_2_H_4_ as an intermediate; here the reduction of NO_2_^−^ -N occurs with four electrons to form hydroxylamine (NH_2_OH), later an enzyme complex bound to the bacterial membrane converts NH_4_^+^–N and NH_2_OH into N_2_H_4_ (Eqs. 4 and 5). Then, the hydrazine is oxidized to N_2_ in the periplasm (Eq. 6) and the electron equivalents are generated, that are transferred through an electron transport chain to NiR in the cytoplasm, site where the reduction of NO_2_– –N to NH_2_OH occurs (Schalk et al. 1998; Kuenen 2008).

4 $${\text{NO}}_{{\text{2}}} ^{ - } + {\text{ 5H}}^{ + } + {\text{ 4e}}^{ - } \to {\text{NH}}_{{\text{2}}} {\text{OH }} + {\text{ H}}_{{\text{2}}} {\text{O}}$$

5 $${\text{NH}}_{{\text{2}}} {\text{OH }} + {\text{ NH}}_{{\text{4}}} ^{ + } \to {\text{N}}_{{\text{2}}} {\text{H}}_{{\text{4}}} + {\text{ H}}_{{\text{2}}} {\text{O }} + {\text{ H}}^{ + }$$

6 $${\text{N}}_{{\text{2}}} {\text{H}}_{{\text{4}}} \to {\text{N}}_{{\text{2}}} + {\text{ 4H}}^{ + } + {\text{ 4e}}^{{ - ~~~~~~~~~~}}$$

In the second, based on genomic, physiological, and biochemical analysis of the species *Ca*.Kuenenia, an alternate catabolism was proposed, which involved not only N_2_H_4_ as an intermediate but also nitric oxide (NO), under three coupled redox reactions (Strous et al. 2006). Here the reaction begins when most nitrite is reduced by using Nir enzymes to NO, using an electron Eq. (7). Next, the enzyme HZS, using three electrons, catalyzes the formation of N_2_H_4_ by using NO as the terminal electron acceptor to oxidize ammonium Eq. (8). Finally, the enzyme HDH oxidizes N_2_H_4_ to N_2_ Eq. (9) releasing four electrons that are available to start the process again, that is, the reduction of NO_2_– –N (one-electron) and the synthesis of N_2_H_4_ in the cytoplasm (three-electrons), thus completing the catabolic cycle of the bacterium and forming a proton gradient across the anammoxosomal membrane, which also provides the energy that is used to drive the synthesis of ATP that energizes the cell (Kartal et al. 2011; Kartal and Keltjens 2016).

7 $${\text{NO}}_{{\text{2}}} ^{ - } + {\text{ 2H}}^{ + } + {\text{ e}}^{ - } \to {\text{NO }} + {\text{ H}}_{{\text{2}}} {\text{O}}$$

8 $${\text{NO }} + {\text{ NH}}_{{\text{4}}} ^{ + } + {\text{ 2H}}^{ + } + {\text{ 3e}}^{ - } \to {\text{N}}_{{\text{2}}} {\text{H}}_{{\text{4}}} + {\text{ H}}_{{\text{2}}} {\text{O}}$$

9 $${\text{N}}_{{\text{2}}} {\text{H}}_{{\text{4}}} \to {\text{N}}_{{\text{2}}} + {\text{ 4H}}^{ + } + {\text{ 4e}}^{ - }$$

**Fig. 1:**
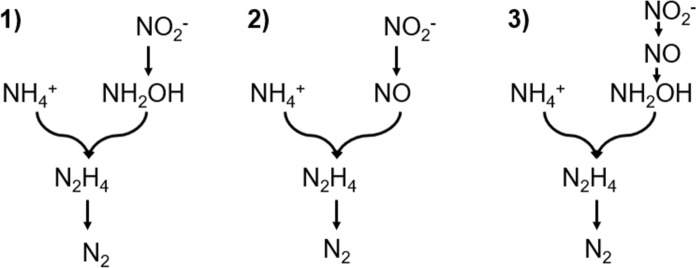
Catabolic scheme proposed by Strous et al. (2006) for the formation of the intermediate N_2_H_4_

Although both are different catabolic pathways, there is general agreement that N_2_H_4_ participates as an intermediate in the catabolism and that NO_2−_ -N is not converted directly to N_2_H_4_, but previously to NH_2_OH or NO. In this sense, Strous et al. (2006) outlined three possible metabolic routes followed by the formation of N_2_H_4_ (Fig. 1), including its production from (1) NH_2_OH, (2) NO and (3) through the presence of NO and NH_2_OH, simultaneously.

## Anabolism

According to van de Graaf et al. (1996), the reducing equivalents necessary to reduce bicarbonate to biomass Eq. (3) are released during the oxidation of NO_2–_–N to NO_3–_-N Eq. (10), a reaction catalyzed by the enzyme NR (Strous et al. 1998).

10 $${\text{NO}}_{{\text{2}}} ^{ - } \to {\text{NO}}_{{\text{3}}} ^{ - } + {\text{ 2H}}^{ + } + {\text{ 2e}}~~$$

**Fig. 2:**
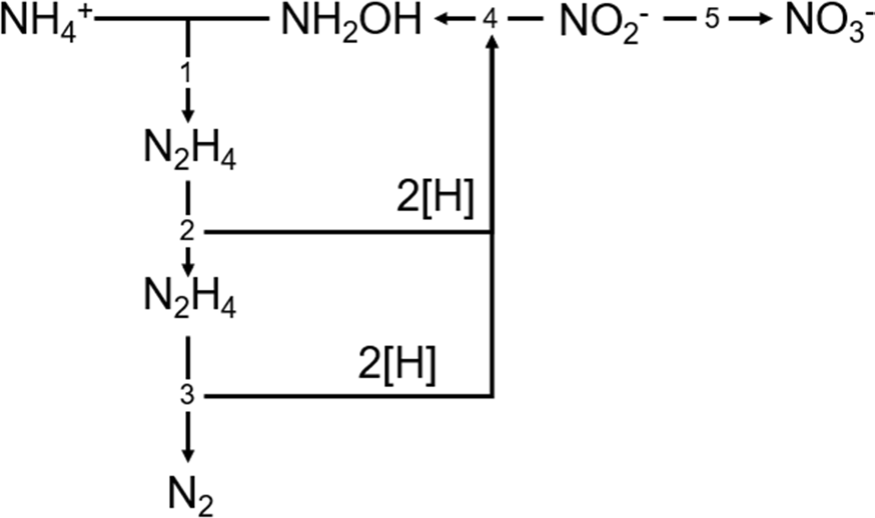
Anammox metabolic pathway that includes nitrate formation proposed by Van de Graaf et al. (1997)

Consequently, biomass growth is associated with nitrate production by oxidizing a small part of nitrite (Eq. 3). Since approximately four moles of nitrite are oxidized for each mole of fixed carbon. The metabolic pathway that involves NO_3_^−^−N production was outlined by van de Graaf et al. (1997) (Fig. 2). First, ammonium is oxidized with hydroxylamine to form hydrazine (Step 1). The reducing equivalents derived from hydrazine reduce NO_2–_ -N to form more NH_2_OH (Steps 2 and 4) and N_2_ (Steps 2 and 3). At the same time, a part of the NO_2_^−^-N is oxidized to nitrate to generate the reducing equivalents necessary for the growth of biomass (Step 5).

## Anaerobic ammonium oxidation coupled to the reduction of alternative electron acceptors

Although nitrite is widely accepted as the main electron acceptor in anammox process, its presence in wastewater rarely occurs at significant levels. Some studies have reported that other electron acceptors, including nitric oxide, sulfate, Fe(III), carbonaceous materials and lately electrodes of BES may support ammonium oxidation under anaerobic conditions. Thus, studying and applying all these processes not only contributes to our understanding of the N cycle and its environmental interactions, but also resolve the problem of industrial wastewater with high nitrogen content and specific pollutants (*e.g*. sulfate) through co-treatment.

## Nitric oxide

Unlike NO_2_^−^-N, nitric oxide (NO) is widely available in water because its formation occurs continuously in the N conversion cycle, it is considered a reactive and toxic compound for certain microorganisms, but not for anammox bacteria (Kartal et al. 2010; Rikmann et al. 2012). To date, only one study has shown that NO may be used as an electron acceptor for NH_4_^+^ oxidation under anaerobic conditions (Hu et al. 2019). Results showed that up to 120 mg NH_4_^+^-N/d can be oxidized with the reduction of 318 mg NO-N/l. The ratio of reduced NO to oxidized NH_4_^+^ was 1.59 (close to the predicted stoichiometry, see Eq. (11). These findings revealed that *Ca*. Kuenenia stuttgartiensis is a NO-dependent anammox bacterium in the absence of nitrite. The use of NO as electron acceptor was also confirmed by transcriptional activity because the proteins involved in nitrite uptake and consumption were transcriptionally down-regulated.

11 $${\text{6NO }} + {\text{ 4NH}}_{{\text{4}}} ^{ + } \to {\text{5N}}_{{\text{2}}} + {\text{ 6H}}_{{\text{2}}} {\text{O }} + {\text{ 4H}}^{ + } ~~~~~~~~~~~~~~~\Delta {\text{G'}}^{{\text{0}}} = {\text{ }} - {\text{1784 kJ}}/{\text{mol}}$$

Reaction 11 indicated that nitrate was not produced, and the only product was N_2_. Lack of nitrate in the effluent refutes the assumption that, under anaerobic conditions, anammox bacteria acquire electrons for reducing equivalents necessary for cell carbon fixation. At least for this species, NO_2_^−^-N seems not to be essential for biomass synthesis. In the case of N_2_O, its accumulation was insignificant and was related to the metabolism of other community members.

Although now there is only one study suggesting that nitric oxide may be used as electron acceptor for NH_4_^+^-N oxidation, investigation and further implementation of this process is required to promote this process at full-scale, which may be suitable to mitigate NO release. Additionally, further studies are needed to elucidate details on the metabolic pathway and the potential interactions between anammox bacteria with other microbial communities.

## Sulfammox

Sulfate-reducing ammonium oxidation (sulfammox) is a biologically mediated process that uses sulfate (SO_4_^2−^) as electron acceptor and NH_4_^+^-N as electron donor to produce N_2_ under anaerobic conditions (Bi et al. 2020). The discovery of the sulfammox has been proposed as a novel link between the N and S biogeochemical cycles (Rios-Del Toro et al. 2018a) and a promising process for the treatment of some industrial wastewater with high concentrations of ammonium and sulfate, such as those derived from effluents from seafood, chemical, textile, paper, fermentation, and sugar production factories (Rikmann et al. 2012, 2016). Although the mechanism of sulfammox is not well understood, Fdz-Polanco et al. (2001) proposed a hypothesis to explain the pathway in which NH_4_^+^-N oxidation is linked to SO_4_^2−^ reduction, with N_2_ and elemental sulfur (S^0^) as reaction products (Eq. 15):

12 $$\text{3SO}_\text{4}^{\text{2}-}+{\text{ 4NH}}_4^+\rightarrow\text{4NO}_\text{2}^-+\text{ 3S}^{{2}-}+{\text{ 4H}}_\text{2}\text{O }+\text{ 8H}^+$$

13 $$\text{ 3S}^{{2}-} + {\text{ 2NO}}_{{\text{2}}} ^{ - } + {\text{ 8H}}^{ + } \to {\text{N}}_{{\text{2}}} + {\text{ 3S}}^{0} + {\text{ 4H}}_{{\text{2}}} {\text{O}}$$

14 $${\text{2NO}}_{{\text{2}}} ^{ - } + {\text{ 2NH}}_{{\text{4}}} ^{ + } \to {\text{2N}}_{{\text{2}}} + {\text{ 4 H}}_{{\text{2}}} {\text{O}}$$

15 $${\text{SO}}_{{\text{4}}} ^{{{\text{2}} - }} + {\text{ 2NH}}_{{\text{4}}} ^{ + } \to {\text{S}}^{0} + {\text{ N}}_{{\text{2}}} + {\text{ 4H}}_{{\text{2}}} {\text{O}}~~~\Delta {\text{G}} = - {\text{46 kJ}}/{\text{mol}}$$

The overall sulfammox reaction occurs in three consecutive biochemical reactions. Initially, NH_4_^+^-N is oxidized to nitrite inside the bacterial cell while sulfate is reduced to sulfide (S^2−^) (Eq. (12)). Subsequently, part of the nitrite is reduced by sulfide with nitrogen gas and elemental sulfur as terminal products (Eq. 13). Finally, the remaining fraction of nitrite that was not used continues its conversion to nitrogen gas through the conventional anammox (Eq. 14).

Schrum et al. (2009), on the other hand, suggested that other mechanisms were involved in the simultaneous removal of NH_4_^+^-N and SO_4_^2−^ under the same environmental conditions. Based on this assumption, a higher NH_4_^+^-N/SO_4_^2−^ ratio promotes the formation of sulfide (HS^−^) (Eq. 18). Firstly, NH_4_^+^-N oxidation to NO_2_^−^-N occurs while sulfate is reduced to HS^−^ (Eq. (16), and then the nitrite reacts with NH_4_^+^-N via the traditional anammox (Eq. 17).

16 $${\text{3SO}}_{{\text{4}}} ^{{{\text{2}} - }} + {\text{ 4NH}}_{{\text{4}}} ^{ + } \to {\text{4NO}}_{{\text{2}}} ^{ - } + {\text{ 3HS}}^{ - } + {\text{ 4H}}_{{\text{2}}} {\text{O }} + {\text{ 5H}}^{ + }$$

17 $${\text{NO}}_{{\text{2}}} ^{ - } + {\text{ NH}}_{{\text{4}}} ^{ + } \to {\text{N}}_{{\text{2}}} + {\text{ 2H}}_{{\text{2}}} {\text{O}}$$

18 $${\text{3SO}}_{{\text{4}}} ^{{{\text{2}} - }} + {\text{ 8NH}}_{{\text{4}}} ^{ + } \to {\text{3HS}}^{ - } + {\text{ 4N}}_{{\text{2}}} + {\text{ 12H}}_{{\text{2}}} {\text{O }} + {\text{ 5H}}^{ + } ~~~\Delta {\text{G}}^{\prime0} = {\text{ }} - {\text{22 kJ}}/{\text{mol}}$$

In this process, HS^−^ production is associated with the presence of organic substrates (“CH_2_O”) in organotrophic environments, because sulfate conversion is attributed to the heterotrophic sulfate reduction by organic matter (Eq. 21). This pathway involves the NH_4_^+^-N oxidation to NO_3_^−^-N using sulfate as electron acceptor (Eq. 19) coupled to heterotrophic denitrification (Eq. 20). In fact, Dominika et al. (2021), highlighted the importance of the sulfide-dependent autotrophic denitrification during the process because without any component, sulfammox cannot occur.

19 $${\text{SO}}_{{\text{4}}} ^{{{\text{2}} - }} + {\text{ NH}}_{{\text{4}}} ^{ + } ~ \to {\text{NO}}_{{\text{3}}} ^{ - } + {\text{ HS}}^{ - } + {\text{ H}}_{{\text{2}}} {\text{O }} + {\text{ H}}^{ + }$$

20 $$5''{\text{CH}}_{2} {\text{O}}'' + 4{\text{NO}}_{3} ^{ - } + 4{\text{H}}^{ + } \to 5{\text{CO}}_{2} + 2{\text{N}}_{2} + 7{\text{H}}_{2} {\text{O}}$$

21 $$4{\text{SO}}_{4} ^{2 - } + {\text{ 4NH}}_{{\text{4}}} ^{ + } + {\text{5}}''{\text{CH}}_{{\text{2}}} {\text{O}}'' \to {\text{4HS}}^{ - } + {\text{2N}}_{{\text{2}}} + {\text{ 5CO}}_{{\text{2}}} + {\text{ 11H}}_{{\text{2}}} {\text{O}}$$

According to Yang et al. (2009), a pure chemical reaction between ammonium and sulfate under abiotic conditions is not possible. In this regard, it is important to know which microorganisms are involved in this biological process to glimpse the possible metabolic pathways. So far, the knowledge regarding microorganisms is still limited because only two functional bacteria capable of actively participating in the process have been described. The first, is an anammox functional bacterium isolated and sequenced more than a decade ago, named *Ca.* Anammoxoglobus sulfate, able of oxidizing NH_4_^+^-N into NO_2_^−^-N (Liu et al. 2008). The second one, *Bacillus benzoevorans*, a bacterium isolated from a laboratory-scale reactor that simultaneously removed ammonium and sulfate (Cai et al. 2010). However, other microbes, such as *Planctomycetes, Verrucomicrobia, Sulfurimonas, Desulfuromonadales, Desulfovibrio, Desulfuromonas, Desulfurobulbus*, *Rhodobacteraceae* and *Thiobacillus* have been reported as potentially involved in the sulfammox process (Rikmann et al. 2016; Prachakittikul et al. 2016; Wang et al. 2017; Rios-Del Toro et al. 2018a; Qin et al. 2021).

Although the sulfammox process has received wide attention over the last few years, the knowledge regarding metabolic pathways, microbial community structure and its interactions is still limited. Besides, the genomic and physiological evidence for putative enzymes involved in the sulfammox process has not yet been identified. Therefore, molecular microbiological tools must be implemented to identify functional microorganisms and their roles in N cycle (Liu et al. 2021).

## Feammox

Ferric iron reduction coupled to anaerobic ammonium oxidation (feammox) is a ferric iron-dependent autotrophic process for biological nitrogen removal (Hu et al. 2022). Its relevance is the connection between the biogeochemical cycles of Fe and N, which contributes to nitrogen loss in ecosystems as diverse as paddy soils, wetland soils, riparian zones, anaerobic tropical soils, agricultural drainage ditches, eutrophic lakes, and marine sediments (Shrestha et al. 2009; Wang et al. 2018; Rios-Del Toro et al. 2018a; Ding et al. 2021). Feammox is a type of extracellular respiration because, as solid electron acceptors, Fe(III) oxides cannot diffuse into the cells to receive electrons from NH_4_^+^-N oxidation to reduce ferric iron (Sun et al. 2023).

In this process, Fe(OH)_3_ or other Fe(III) oxides act as electron acceptor to oxidize NH_4_^+^-N and produce N_2_, NO_2_^−^-N and NO_3_^−^-N (Eqs. 22, 23, 24), according to the energy required. Ammonium oxidation to N_2_ (−245 kJ/mol) is thermodynamically more favorable than the NH_4_^+^-N oxidation to NO_2_^−^-N (−164 kJ/mol) and to NO_3_^−^-N (−207 kJ/mol) (Zhao et al. 2022). Kim et al. (2002), on the other hand, stated that the formation of the products is highly pH dependent. Equation (22) could occur under a wide range of pH, while Eqs. (23) and (24) can only exist below pH 6.5 (Yang et al. 2012). In summary, feammox yields less energy to oxidize ammonium and could be applied to a wide pH range as compared to the traditional anammox (Puyol et al. 2014; Ren et al. 2020).

22 $${\text{3Fe}}\left( {{\text{OH}}} \right)_{{\text{3}}} + {\text{ 5H}}^{ + } + {\text{ NH}}_{{\text{4}}} ^{ + } \to {\text{3Fe}}^{{{\text{2}} + }} + {\text{9H}}_{{\text{2}}} {\text{O }} + {\text{ }}0.{\text{5N}}_{{\text{2}}} ~~~~~\Delta {\text{G}}^{\prime0} = {\text{ }} - {\text{245 kJ}}/{\text{mol}}$$

23 $${\text{6Fe}}\left( {{\text{OH}}} \right)_{{\text{3}}} + {\text{ 1}}0{\text{H}}^{ + } + {\text{ NH}}_{{\text{4}}} ^{ + } \to {\text{6Fe}}^{{{\text{2}} + }} + {\text{ 16H}}_{{\text{2}}} {\text{O }} + {\text{ NO}}_{{\text{2}}} ^{{ - ~~~~~~~~}} \Delta {\text{G}}^{\prime0} = {\text{ }} - {\text{164 kJ}}/{\text{mol}}$$

24 $${\text{8Fe}}\left( {{\text{OH}}} \right)_{{\text{3}}} + {\text{ 14H}}^{ + } + {\text{ NH}}_{{\text{4}}} ^{ + } \to {\text{8Fe}}^{{{\text{2}} + }} + {\text{ 21H}}_{{\text{2}}} {\text{O }} + {\text{ NO}}_{{\text{3}}} ^{{ - ~~~~~~~~}} \Delta {\text{G}} ^{\prime0} = {\text{ }} - {\text{2}}0{\text{7 kJ}}/{\text{mol}}$$

Until now, the biochemical mechanisms of NH_4_^+^-N oxidation in feammox have not been fully revealed, but three pathways to reduce Fe(III) by feammox microorganisms have been suggested. Among them, direct electron transfer occurs when microorganisms and iron oxides are in direct contact through the cell membrane or, more precisely, a redox-active membrane organelle (*e.g*., cytochromes), forming an extracellular electron transfer pathway between the intracellular respiratory chain and external iron (Harnisch et al. 2011; Xia et al. 2022). Indirect electron transfer via exogenous compounds happens once redox active electron shuttles (*e.g*., biochar, humic substances, activated carbon, and quinones) reversibly carry the electrons released by ammonium oxidation and transfer them to solid Fe(III) oxides, resulting in their microbial reduction (Xia et al. 2022; Sun et al. 2023). The last mechanism of Fe(III) reduction is via chelating agents or protein nanowires, in which the chelating agent promotes the iron dissolution and facilitates the interactions between Fe(III) oxides and the microbes (Chakraborty et al. 2011). The last mechanism implies that protein nanowires carry out a long-range electron transfer to Fe(III) oxides by conductive filaments synthesized by microorganisms (Malvankar and Lovley 2014; Xia et al. 2022; Sun et al. 2023).

Feammox has attracted more attention in exploring its basic principle and identifying its microorganisms over the last few years. So far, it has been reported that two types of functional bacteria, *Acidimicrobiaceae sp. A6* (Ruiz-Urigüen et al. 2018) and a new strain FC61, *Klebsiella sp.* (Su et al. 2016), can oxidize ammonium by using ferric iron as electron acceptor under anaerobic conditions (Zhao et al. 2022). Even though both strains have been isolated, their functional genes and enzymes have not been identified. Because of the limited information regarding functional isolated strains, investigations have shown that some iron-reducing bacteria (IRB) may be involved in the feammox reaction, including *Nitrososphaeraceae*, *Pseudomonas, Geobacter spp. *(Li et al. 2019b), *Shewanella spp.*(Newsome et al. 2018), *Acidobacteria, Bacteroidetes* (Ding et al. 2017), *Fervidicella* (Yao et al. 2020), *Anaerospora, Comamonadaceae* (Bao and Li 2017), *Actinomarinales, Microbacteriaceae, Pseudonocardiaceae, Nocardiopsaceae, Eggerthellaceae* (Rios-Del Toro et al. 2018a), *Anaeromyxobacter, Desulfosporosinus, Decloromonas* and *Geothrix* (Zhou et al. 2016a, b).

In the case of *Geobacter spp*. and *Shewanella spp*, it has been proposed that both may play an important role in the feammox process, based on the positive correlations among: (i) the abundance of these taxa, (ii) the rates of Fe(III) reduction, and (iii) the rates of ^30^N_2_ production (Li et al. 2015). Apart from IRB, some species of anammox bacteria (including *Ca.* Kuenenia stuttgartiensis, *Ca.* Scalindua wagneri, *Ca.* Brocadia sinica *and Ca.* Brocadia fulgida) have been associated with the reduction of Fe(III) to Fe(II) and the loss of nitrogen using organic matter and NO_2_^−^-N as electron donor and electron acceptor, respectively (Zhao et al. 2014; Li et al. 2018; Hu et al. 2022; Xia et al. 2022).

So far, some potential feammox microbes have been reported. However, the microbial community structure is still unclear. Besides, little is known about the metabolic pathway and the mechanisms of NH_4_^+^-N oxidation because it is difficult to explain the metabolism and physiology at the level of organism, based on enrichment experiments in mixed cultures. Hence, the study of genomes involved in the process through metagenomic approaches is crucial in the future to provide a comprehensive overview of the microbial community that promotes feammox, infer its metabolic potential, suggest putative electron transfer routes, and explore the potential roles of the community members and the interspecies interactions (Tan et al. 2022).

## Humic substances (NOM-ANAMMOX)

As the most typical electron shuttle in nature, humic substances (humics) are heterogeneous, high-molecular weight, organic materials that represent the main redox fraction of natural organic matter (NOM) in soils, sediments, and aquatic environments (Lovley et al. 1996: Sun et al. 2022). Humics have been shown to act as electron acceptors during microbial respiration (Valenzuela et al. 2020). Microbial electron transfer to humics could be significant in environments even at low concentrations of humics (Scott et al. 1998). Quinone moieties in humics are the most important electron-accepting and shuttling groups for microbial extracellular respiration (Wolf et al. 2009). Thus, quinone groups have been considered as “humus-like” to study their participation as electron acceptors for respiration, redox mediators for reduction processes, and as electron donors to microorganisms (Field et al. 2000; Wang et al. 2018).

Under anaerobic conditions, humics drive nitrogen loss through different pathways. Firstly, they serve as electron acceptor during NH_4_^+^ oxidation (Rios-Del Toro et al. 2018b). In addition, their reduced form (*e.g.*, hydroquinones) act as electron donor for the reduction of nitrogen species (*e.g.*, nitrate or N_2_O) and finally, they can be used as redox mediators for accelerating electron transport from cells to the final electron acceptor (Aranda-Tamaura et al. 2007; van der Zee and Cervantes 2009; Valenzuela et al. 2020).

Despite the versatility of humics and their quinone analogues, their application in the anammox process has been poorly investigated. Qiao et al. (2014) demonstrated that model quinone compounds (AQDS, LAW and AQC) increased the enzymatic activity of anammox biomass. In another study, Zhou et al. (2016a, b) reported that AQDS improved the production rates of ^30^N_2_ and ^29^N_2_, as well as Fe(II) production, which potentially increased the feammox-mediated nitrogen loss up to 340%. Rios-Del Toro et al. (2018b) provided the first direct evidence that quinone moieties in NOM can serve as electron acceptor to drive anammox by the microbial community in marine sediments. Using stoichiometry and spectroscopy, they demonstrated the microbial reduction of NOM coupled to anaerobic NH_4_^+^-N oxidation. The overall reaction for this NOM-dependent anammox process is given below (Eq. 25). The results showed that N_2_ production rates were three times higher than the performance achieved in experiments without exogenous NOM.

25 $${\text{NH}}_{{\text{4}}} ^{ + } + {\text{ 1}}.{\text{5Q}} - {\text{NOM}}_{{{\text{ox}}}} \to 0.{\text{5N}}_{{\text{2}}} + {\text{ 1}}.{\text{5QH}}_{{\text{2}}} - {\text{NOM}}_{{{\text{red}}}} + {\text{ 4H}}^{ + } ~~~~~\Delta {\text{G}}^{\prime0} = {\text{ }} + {\text{5}}.{\text{8 to }} - {\text{124 kJ}}/{\text{mol}}$$

This reaction displays the energy yield obtained for the whole range of redox potential reported for NOM (from − 300 mV to + 150 mV, Rios-Del Toro et al. 2018b). Where Q-NOM_ox_ refers to quinone equivalents (2 electron equivalents per quinone moiety) and QH_2_-NOM_red_ represents quinone equivalents accumulated as reduced NOM.

Finally, Dey et al. (2021) demonstrated a promotion in the biological nitrogen fixation by adding humin (a type of humics) as a redox mediator in an anaerobic consortium. Tests using intact and oxidized humin did not show any change in nitrogenase activity, which suggests that humin was used as a redox mediator rather than as a carbon source for cellular metabolism. Although, in this research, the NOM was not involved in the anammox process, its environmental relevance is indisputable since the evidence shows that NOM plays an active role in N cycle, both in mechanisms of loss and fixation of nitrogen.

The evidence suggests that humics and their quinone analogues support the anoxic oxidation of ammonium. To date, no functional bacteria have been reported to play a key role in ammonium oxidation with NOM as electron acceptor. However, taxonomic characterization shows that most predominant bacterial phylotypes detected during the NOM-dependent anammox processes are associated with *Phycisphaeraceae*, *Moraxellaceae, Actinomarinales*, *Acidiferrobacteraceae*, *Acetobacteraceae, Rhodobacteraceae*, *Anaerolineaceae, Acidithiobacillaceae, Pelobacteraceae*, *Desulfovibrio*, and some denitrifiers (Qiao et al. 2014; Rios-Del Toro et al. 2018b; Dey et al. 2021). Additionally, *Nitrosopumilaceae* (an archaeal family) has been highly enriched during this process (Rios-Del Toro et al. 2018b). In terms of anammox bacteria, *Ca.* Kuenenia, *Ca.* Brocadia and *Ca.* Jettenia are the dominant populations in the presence of humics (Qiao et al. 2014; Rios-Del Toro et al. 2018b; Dey et al. 2021). However, further investigations related to the NOM-anammox process are necessary, to characterize and isolate the potential functional bacteria, genes, and enzymes to expand our understanding of this process.

## Graphene oxide

Graphene oxide (GO) is a derivative of graphite, composed of monolayers of carbon atoms and oxygenated functional groups (*e.g*., hydroxyl, epoxy, carbonyl, and carboxyl) that can donate and accept electrons, which can mediate microbial extracellular electron transport (Colunga et al. 2015; Toral-Sánchez et al. 2017). Because of their exceptional electron transfer ability, research in wastewater treatment has focused on the application of GO and its reduced form (rGO) to promote efficient nitrogen removal, particularly by anammox. Indeed, the electron transfer capacity in rGO appears to be about three orders of magnitude higher than that of GO (Yin et al. 2016b). In 2013, batch experiments showed that GO promote NH_4_^+^ oxidation due to an improved anammox enzyme activity and a high secretion of EPS, which facilitated the adhesion bacteria to the surface of GO (Wang et al. 2013). To date, it is known that EPS provide nutrition to microorganisms, facilitate their development as high-size macroflocs, improve the retention of biomass, and develop resistance to negative environmental factors (Suárez-Iglesias et al. 2017; Wells et al. 2017).

Years later, it was found that the nitrogen removal efficiency was improved up to 17% after adding the optimal dose of 100 mg/l of GO to anammox systems, which was rapidly convert to rGO (Yin et al. 2015a, b). Therefore, the activities of hydrazine hydrolase, nitrite reductase and nitrate reductase were stimulated up to 2.7 times due to the acceleration of electron transfer rate (Yin et al. 2015a; Zhang et al. 2022). Increase in the enzyme activity due to GO remained even at low temperatures (Wang et al. 2014; Tomaszewski et al. 2019). Improvements following the addition of GO and rGO are of utmost importance since acceleration of the activity of anammox bacteria is also reflected by a decrease on the lag phase. Recently, Shaw et al. (2020) demonstrated that GO can act as electron acceptor for sustaining the anammox process by EET. The results showed that without nitrite and nitrate in the synthetic medium, some anammox bacteria (*Ca.* Brocardia and *Ca.* Kuenenia) could transfer electrons to GO. It was the first study that suggested that anammox bacteria have EET capability, because they oxidized NH_4_^+^-N to N_2_ using the insoluble material as electron acceptor and formed rGO. The latter was proposed after the medium changed to a dark black color (due to the formation of insoluble precipitates). Wang et al. (2013) also observed a change in the color of synthetic wastewater, due to the reduction of GO by the anammox microorganisms. This hypothesis was subsequently confirmed by Raman spectroscopy analysis. Furthermore, given the size of GO sheets, its internal incorporation into the cells was not possible. Thus, extracellular electron transfer can only explain its reduction to rGO.

## Anammox in bioelectrochemical systems

Recently, ammonium removal in BES has been proposed as an alternative to conventional nitrogen-removing systems for wastewater treatment. In this technology, nitrogen is removed from wastewater coupled to electricity generation in a MFC or hydrogen production in a MEC (Katuri et al. 2019). Ammonium is considered a potential fuel for SBE because (1) it is a major pollutant in wastewaters, (2) its oxidation exhibits a negative Gibss free energy (−357 kJ/mol), indicating that it is a spontaneous reaction that occurs without external energy input, and (3) during its oxidation, ammonium releases a large number of electrons, which can be used to synthesize ATP, produce electricity or promote specific reduction reactions in the cathodic chamber (He et al. 2009; Xie et al. 2013; Zhan 2020). Although, in MFC and MEC, there is a constant interaction between microorganisms and the electrodes (electron acceptor), each one presents differences in its operation and reaction products, which are detailed below.

**Fig. 3:**
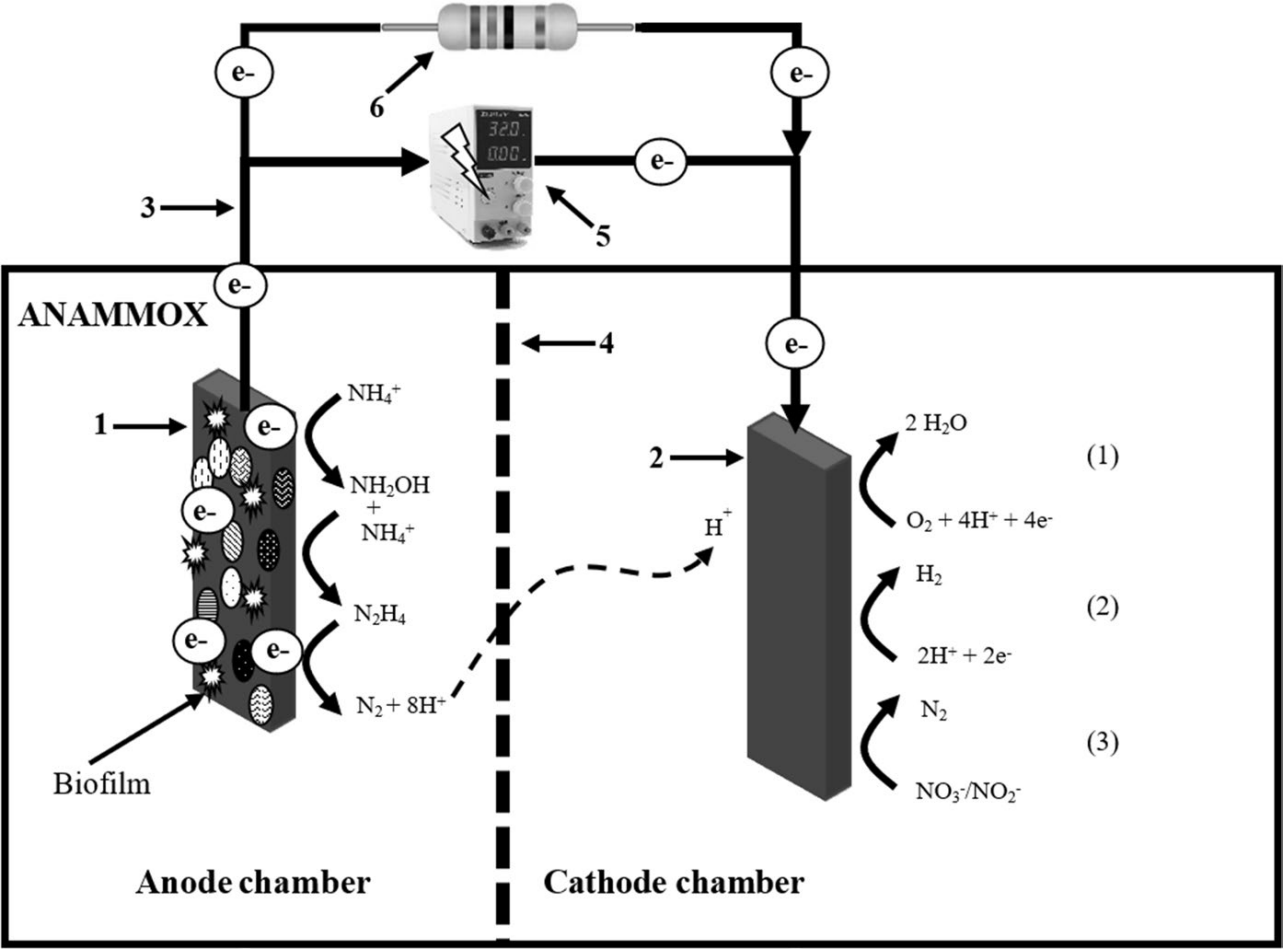
Schematic diagram of a MFC and a MEC coupled to anammox. The anode chamber illustrates the anammox reaction using the electrode as electron acceptor, considering the mechanisms reported by Shaw et al. (2020). The cathode chamber shows potential reactions that can take place: water production (1), hydrogen production (2) and denitrification (3)

MFC is a promising system for concurrent wastewater treatment and electricity generation by using microorganisms as catalysts to oxidize ammonium and convert its chemical energy into electrical energy (Kong et al. 2022). In these BES, the reaction occurs spontaneously, releasing energy that can be used as an electrical source (Li et al. 2016). Figure 3 illustrates the coupling of MFC and anammox: (1) anode used as electron acceptor for direct NH_4_^+^-N oxidation by EAB (Zhan 2020), (2) cathode used as electron donor to drive reduction reactions (Kokabian et al. 2018), (3) external circuit, which is responsible for the adequate passage of electrons collected from the anode to the cathode (Oliot et al. 2016), (4) selective proton membrane used as an electrolyte to transport protons from the anode to the cathode, which separates the two chamber (Zhang et al. 2013), and (6) external resistance that is used to determine the electron flow rate in the MFC (Zhou et al. 2022). Regarding its operation, electrons released during NH_4_^+^-N oxidation are collected at the anode (anodic-anammox) and transferred to the cathode by the external circuit. In the cathode chamber, there is an oxidizing compound, typically oxygen, which reacts with the protons coming from the anode chamber to form water as a by-product (Zekker et al. 2020).

On the other hand, in a microbial electrolysis cell (MEC), the difference between the anode and the cathode potentials is insufficient, therefore an external power supply is necessary to induce nonspontaneous reactions or to accelerate spontaneous reactions (Li et al. 2016). Recently, the coupling of MEC-anammox has been proposed as a novel bioelectrochemical approach for nitrogen removal because, although the ammonium oxidation in MFC has been confirmed, the potential difference between the electrodes was not sufficient, so that a small power should be supplied to facilitate NH_4_^+^-N oxidation (Koffi and Obake 2021). In terms of configuration and operation, MEC are similar to MFC (Fig. 3). However, the external power supply (5) is an important feature of this technology. Ammonium oxidation takes occurs in the anode chamber, the electrons derived from NH_4_^+^-N oxidation are captured by the anode and then transferred to the cathode through the external circuit and released in the cathode chamber to promote specific reduction reactions (*e.g*., reduction of nitrite and nitrate, or protons, to form nitrogen gas or hydrogen, respectively).

## Microbial fuel cells

Theoretically, the anammox reaction has a negative Gibbs free energy of −357 kJ/mol under standard conditions (at pH 7.0, temperature of 25 °C, and air pressure of 1 atm) (Gao and Tao 2011), which is superior to the one reported for the aerobic oxidation process (− 235 kJ/mol), which makes ammonium an appropriate substrate for BES, that is, serving as an electron donor. In addition, ammonium has the lowest oxidation state (− 3) of nitrogen, therefore the number of electrons released during its oxidative reaction is higher; thus, ammonium can also serve as a substrate for other electroactive bacteria (Jetten et al. 2001; Xie et al. 2013).

MFC coupled to the anammox process for simultaneous nitrogen removal and power generation are emerging as promising technologies in the last years (Kong et al. 2022). Researchers have made great efforts on nitrogen removal by applying this coupling. He et al. (2009) were among the first to report anammox-dependent electricity production at a MFC. They proved that adding ammonium along with NO_2_^−^-N and NO_3_^−^-N to the BES increased current production (up to 0.078 mA). Batch experiments showed that removing NH_4_^+^-N from the feeding medium resulted in the interruption of current production, which confirmed that ammonium was the main supplier of electrons in the MFC. In addition, by means of control experiments with different salts of ammonium in its chemical structure, it was ruled out that the current production was the result of the increase in the ionic strength of the medium. Molecular analysis based on 16 S rRNA gene sequencing revealed that aerobic ammonium oxidizing bacteria and denitrifying bacteria were found on the surface of the electrodes, whereas no species associated with anammox-type bacteria were detected. The nitrogen removal efficiency of the BES was 69%, while the coulombic efficiency was only 0.34%. Lee et al. (2013) also observed simultaneous electricity production and nitrogen removal in an anammox biocathode MFC. The results showed increases of more than 50% in the power density (12 mW/m^2^) and nitrogen removal efficiency greater than 90% (0.055 kgN/m^3^∙d). In the same year, Xie et al. (2013) also achieved high ammonium conversion to nitrogen gas (~ 100%) and power densities of 9.7 mW/m^2^ in a MFC. However, the results were associated with the presence of dissolved oxygen, which contributed to the distribution of electrons through the system.

To date, the maximum power density reported for an MFC linked to anammox is 172.2 mW/m^2^, in which this system was operated with anaerobic digestate as inoculum (which also served as a carbon source for biomass synthesis). However, the power density was associated with the presence of intrinsic organic matter in the digestate, which explained the colonization of *G. sulfurreducens* (electrogenic bacteria) on the surface of the electrode and the removal of the organic carbon content by up to 60%. Nitrogen was removed by 40%, and *Ca*. Brocadia anammoxidans was identified as a member of the microbial community that played a role in nitrogen removal (di Domenico et al. 2015). Hassan et al. (2018) applied a MFC for energy generation from landfill leachate treatment using anammox sludge to promote NH_4_^+^ oxidation. In the anodic chamber, ammonium was used as fuel during anammox process because an increase of up to 240 mg/l of ammonium, promoted an enhancement in the efficiency of the MFC. After finishing the experiments, ammonium and nitrite were removed from the medium by 66% and 86%, respectively. Sequencing analysis showed that in addition to anammox bacteria, denitrifying bacteria (of the phylum *Proteobacteria*) and electrogenic bacteria (*Geobacter sp*) were abundant in the microbial community of the anode.

## Microbial electrolysis cells

The first application of a MEC coupled to the anammox process was reported by Zhan et al. (2012), whose research consisted in designing a single compartment microbial cell to evaluate the effect of low voltage application on ammonium removal rate. The MEC contained an anodic and a cathodic electrode, on which nitrifying and denitrifying biofilms were enriched, respectively. The data showed that the potential difference between the electrodes in these BES was deficient, so that a small power (< 0.8 V vs. SHE) was supplied externally using a direct current power supply to facilitate NH_4_^+^-N oxidation. After increasing the voltage from 0.2–0.4 V vs. SHE in the anode, the nitrogen removal rate and the electrical current increased from 70–92% and from 4.4–14 mA, respectively, while the coulombic efficiency was 94%. The explanation for the process improvement is because the increase in voltage causes a change in the electrode potential, so that it is more capable of receiving or transferring charges (electrons). In this case, the electrons released from NH_4_^+^-N are used for NO_2_^−^-N and NO_3_^−^-N reduction in the cathode.

Another investigation showed that in the absence of nitrite, the anode served as the electron acceptor in a dual chamber electrolysis cell. Analytical methods showed that nitrate and nitrite (at low concentrations) were the main products of NH_4_^+^ oxidation. 16 S ribosomal RNA analysis defined *Nitrosomonas europaea*, and phylotypes from the genera *Empedobacter*, *Comamonas* and *Paracocus* as the main members of the enriched microbial community on the surface of the electrode, which explained the accumulation of nitrate (Qu et al. 2014).

Similarly, Zhang et al. (2014) proposed the anode as the electron acceptor in a two-chamber MEC. The results showed that by applying a voltage of 0.8 V vs. SHE in the anode, ammonium was removed up to 83% and the current production was increased three times, with respect to lower applied voltages (0 and 0.4 V). At the end, NO_3_^−^-N accumulation confirmed the prevalence of nitrification, due to the contamination of autotrophic microorganisms in the cathodic compartment. The microbial community was dominated by *Nitrosomonas europaea*. Two additional investigations coupled anammox to a MEC. In the first investigation, Zhu et al. (2016) emphasized that controlling the anodic potential in a BES increases the ammonium removal efficiency as compared to a conventional anammox reactor without electrodes because the efficiency of the process increased by at least 29% once a given anode potential was set at 0.2 V vs. SHE. Independent experiments confirmed the electrochemical removal mechanism of the process once the anodic potential was excluded because the nitrogen removal declined to the level observed in the control reactor. By means of scanning electron microscopy and FISH molecular analysis, the abundance of anammox bacteria (56%) and aerobic ammonium-oxidizing bacteria adhered to the surface of the anode was confirmed. This is an important evidence because it suggests that anammox bacteria are electrically active. In the second study, Li et al. (2016) designed a BES made up of a MEC and a MFC; the latter was conceived to remove the organic load and to provide the voltage required by the MEC. Batch tests showed that at least 85% of the nitrogen was removed from the system during the first 10 days of operation, which represented a higher efficiency as compared to the 62% achieved by the conventional anammox process. Further evidence showed that anammox efficiency can be increased by applying direct current to the process, especially through increasing the key enzymatic activity in anammox bacteria and facilitating substrate turnover, which achieved better nitrogen removal efficiency (Yin et al. 2015b, 2016a).

In a similar way, Vilajeliu-Pons et al. (2018) observed that with an applied anode potential of + 0.8 V vs. SHE, in a dual-chamber MEC, hydrogen production was favored in the cathodic compartment and ammonium removal rates were comparable to those achieved with conventional anammox, but with lower energy consumption (35 times lower) and without accumulation of nitrite, nitrate or nitric oxide. Nitrogen isotopic labelling revealed that hydroxylamine was the main intermediate of ammonium electrochemical oxidation. Furthermore, phylogenetic analysis revealed the presence of nitrifying-, anammox- (Brocardia and Kuenenia), denitrifying-, feammox- and *Firmicutes* phylum in the anode biofilm of the dual-chamber BES reactor.

Recently, anammox bacteria have been reported to transfer extracellular electrons for ammonium oxidation to nitrogen gas with an electrode of a MEC as the sole electrode acceptor. In this study, Shaw et al. (2020) clearly demonstrated that BES enriched with *Ca.* Brocardia, *Ca.* Scalindua and *Ca.* Kuenenia promoted successful NH_4_^+^-N oxidation and generation of current without nitrite as electron acceptor. The maximum current production was observed with an applied anode potential of + 0.6 V vs. SHE. Isotopic labeling experiments showed that hydroxylamine and hydrazine were the intermediates in the process, while nitrous oxide was not detected. Furthermore, nitrite and nitrate concentrations always remained below the detection limit using the working electrode as the sole electron acceptor, suggesting that both compounds did not play a significant role in the BES. The reactions proposed by the researchers are presented below (Eqs. 26, 27, 28, 29). This mechanism begins with the NH_4_^+^-N oxidation to NH_2_OH. Next, the remaining fraction of NH_4_^+^-N reacts with NH_2_OH to form hydrazine. Finally, the N_2_H_4_ is oxidized to N_2_.

26 $${\text{NH}}_{{\text{4}}} ^{ + } + {\text{ H}}_{{\text{2}}} {\text{O}} \to {\text{NH}}_{{\text{2}}} {\text{OH }} + {\text{ 3H}}^{ + } + {\text{ 2e}}^{ - }$$

27 $${\text{NH}}_{{\text{4}}} ^{ + } + {\text{ NH}}_{{\text{2}}} {\text{OH}} \to {\text{N}}_{{\text{2}}} {\text{H}}_{{\text{4}}} + {\text{ H}}_{{\text{2}}} {\text{O }} + {\text{ H}}^{ + }$$

28 $${\text{N}}_{{\text{2}}} {\text{H}}_{{\text{4}}} \to {\text{N}}_{{\text{2}}} + {\text{ 4H}}^{ + } + {\text{4e}}^{ - }$$

29 $${\text{2NH}}_{{\text{4}}} ^{ + } \to {\text{N}}_{{\text{2}}} + {\text{ 8H}}^{ + } + {\text{ 6e}}^{ - }$$

The dependence of the bacteria on the electrodes was confirmed by different control experiments, which included the addition of inhibitors of specific enzymes, under the operation of the system in open and closed circuits, carrying out experiments with sterile biomass and excluding ammonium from the feeding medium. All tests confirmed that the current production was associated with a biotic reaction. During the experiments, the magnitude of current production was proportional to the ammonium concentration, that is, when ammonium was depleted, current production ceased, but restarted after ammonium was reintroduced.

In the most recent study, Koffi and Obake (2020) anoxic NH_4_^+^-N oxidation and TN removal rates were determined at various applied voltages (0–1.2 V), provided by a MFC. Maximum ammonium removal rate (151 g NH_4_^+^-N/m^3^d) and TN removal rate (95 g-TN/m^3^∙d) without aeration at the cell applied voltage of 0.8 V. In addition, by means of the operation of the cell in open and closed circuit, the bioelectrochemical nature of process was verified (that is, with the anode as an electron acceptor). The production of nitrogen gas as the only product and the absence of nitrite and nitrate in the system confirmed that the anammox process was efficient. In addition, ^15^ N isotope trace experiments and microbial community analysis showed that the most dominant populations detected in the anode belonged to the phyla *Proteobacteria, Bacteroidetes, Actinobacteria, Chloroflexi, Firmicutes* and *Planctomycetes*. Denitrifying heterotrophic and anammox bacteria were associated with the removal of nitrogen. In the case of anammox bacteria, the genus *Ca.* Brocadia was detected in the anode and in the cathode biofilm. Based on this information, the researchers suggest that the anammox process may contribute to ammonium electrochemical oxidation in this MFC supported by a MEC system.

## Nitrogen recovery in bioelectrochemical systems

With the high demand for food from the growing world population, the production of chemical fertilizers has increased by almost 2% annually (Ledezma et al. 2015). Conventionally, fertilizers are made from nitrogen, an essential nutrient for plant growth, but whose presence in soils is limited (van der Hoek et al. 2018). Although the atmosphere is an abundant source of nitrogen (its content is ~ 80%), this is in a stable and non-reactive form (N_2_), which most plants cannot assimilate; therefore, to be accessible to crops, it must be converted into more reactive species, such as NO_3_^−^-N, NO_2_^−^-N or NH_4_^+^-N. This conversion is known as nitrogen fixation and occurs in two ways: in nature, atmospheric nitrogen is transformed into ammonium and nitrate by prokaryotes (Eq. 30) containing nitrogenase (98%) or by atmospheric deposition through electric lightning (2%) (Sengupta et al. 2015). Artificially, the Haber-Bosch process is used to fix atmospheric nitrogen in the form of ammonia (Freguia et al. 2019), by combining N_2_ and H_2_ (Eq. 31) at high temperature (400–600 °C) and high pressure (20–40 MPa). This results in a process, which is so energy-intensive that it demands 1–2% of the world’s electricity consumption (with a typical energy usage of 9–14.2 kWh/kg NH_3_-N) (Maurer et al. 2003; Erisman et al. 2008; Kugler et al. 2014). Furthermore, hydrogen used for this reaction is processed from natural gas or oil directly, resulting in carbon dioxide as a by-product and an electrical cost of approximately 10 kWh/kg NH_3_-N (Nancharaiah et al. 2016).

30 $${\text{N}}_{{\text{2}}} + {\text{ 8H}}^{ + } + {\text{ 8e}}^{ - } + {\text{ 16ATP}} \to {\text{2NH}}_{{\text{3}}} + {\text{ H}}_{{\text{2}}} + {\text{ 16ADP }} + {\text{ 16 Pi}}~~~~~$$

31 $${\text{N}}_{{\text{2}}} + {\text{ 3H}}_{{\text{2}}} \to {\text{2 NH}}_{{\text{3}}}$$

In addition to the remarkable demand for energy destined to produce nitrogen fertilizers, their increasingly high consumption leads to the discharge of substantial amounts of nitrogen into water streams (it is estimated that 11–16% of the flow of anthropogenic nutrients is directed through wastewater facilities) (Billen et al. 2013). Besides, it has been forecasted that amount of ammonium that will end up in domestic wastewater will rise to 35 million tons per year by 2050 (Bodirsky et al. 2014). Thus, the treatment of nitrogen-polluted wastewater is attracting increasing attention as a crucial step in recovering and recycling nitrogen. While nitrogen that enters to the wastewater streams is removed by effective processes, such as N/DN or anammox, most of this nitrogen is converted to nitrogen gas and is lost to the atmosphere instead of being reused by means of biological fixation (van der Hoek et al. 2018).

In this sense, recovering nitrogen from wastewater is considered as a more sustainable approach than removing it, due to the high costs and environmental pollution involved in artificial nitrogen fixation (Kelly and He 2014). Nitrogen recovery in BES has been proposed as a competitive technology to traditional ammonium removal from wastewater streams, taking advantage of the reactions that occur in the electrodes of the systems. Wong et al. (2014) demonstrated that a bioanode could promote BNF using glucose and diazotrophic microorganisms in nitrogen-deficient wastewater. The open circuit operation of the BES helped to remove glucose from the water and to obtain acetate as a key by-product. Once the BES operation was switched to closed circuit, acetate accumulation began to decrease (up to 30 times) suggesting that the biofilm oxidized acetate under nitrogen deficient conditions. Microbial analysis showed that *Clostridium* (an electrochemically active genus capable of fixing N_2_) dominated the electrode-enriched biofilm in abundance (78%), while the rest (22%) comprised other bacterial groups (*Bacilli, Bacteroidia, Alphaproteobacteria, Betaproteobacteria, Gammaproteobacteria* and *Deltaproteobacteria*).

In a similar study, Rago et al. (2019) designed a single chamber microbial electro-synthesis to produce biomass associated with BNF through electrostimulation. The results showed that using a carbon cloth-based cathode under constant polarization (− 0.7 V vs. SHE) promoted the enrichment of nitrogen-fixing autotrophic microorganisms on the surface of the electrode, in addition to increasing cellular synthesis by at least eighteen times. Through metagenomic analysis, nucleotide sequences that encoded different subunits of the nitrogenase complex were related to different archaea and bacteria (potentially involved in nitrogen fixation), which includes *Methanobrevibacter arboriphilus, Ca.* Accumulibacter spp., *Shewanella mangrovi* and *Methylomonas koyamae*.

Xiao et al. (2016) used a three-chamber MFC aimed at simultaneously removing and recovering nitrogen from synthetic wastewater, to use it as a fertilizer, but without energy input. The anodic and cathodic chambers were separated by an intermediate chamber containing selective cationic and anionic exchange membranes, for the migration of ammonium and nitrate, respectively. The system was operated in batch mode, with microorganisms in the anode chamber and with ferric nitrate as electron acceptor in the cathode chamber, the latter also served as a reference to evaluate the efficiency of the system in nitrate recovery. The output voltage ranged from 600–700 mV with an external load of 500 Ω. The data showed that around 47% of the ammonium and 83% of the nitrate were recovered in the central compartment, confirming that the BES design was efficient in simultaneously removing and recovering nitrogen.

Monetti et al. (2019) designed a three-chamber bioelectrochemical system to bioelectroconcentrate nutrients (including nitrogen) from domestic wastewater. The experimental data revealed that the system showed limited nutrient recoveries due to the poor ionic conductivity of the electrolyte, which resulted in low current densities (< 2 A/m^2^). As soon as it was observed, a potentiostat/galvanostat operated in chronoamperometry mode was used to deliver higher current densities, which increased nitrogen removal by 48%, but with the precipitation of calcium and magnesium salts in the anionic membrane. This phenomenon was explained by the non-selective migration of cations in the central compartment caused by precipitation of these salts, which reduced the selective permeability of the membrane. In addition, the application of high current densities (~ 20 A/m^2^) not only impacted the selectivity of the membrane, but also demanded a higher energy consumption and led to higher ohmic losses, causing uncontrolled potentials that promoted the electrolysis of the water and the disintegration of the graphite electrode.

## Comparison of power demand among biological treatment processes for ammonium removal

WWTPs are recognized as large independent energy consumers because the effluent quality relates to significant power input (Gu et al. 2017; Wang et al. 2019). It is estimated that the operation of WWTPs represent 3–5% of the world’s electricity consumption (Foladori et al. 2015; Dai et al. 2019; Ye et al. 2019). The numbers indicate that up to 50% of the total energy supplied in a conventional WWTPs is used to satisfy the aeration demand for carbon and nitrogen oxidation processes (Gude et al. 2015; Ghimire and Gude 2019). Another study suggests that the contribution of biotreatment processes to total electric power consumption may reach up to 80% (Rodziewicz et al. 2022). In fact, it is expected that its consumption will be higher in the future due to the increase in pollutant load and stringent environmental regulations (Wang et al. 2019). Thus, energy efficiency has attracted more attention from an environmental and economic point of view (Longo et al. 2016). Hence, developing and implementing efficient, energy-producing processes that reduce aeration costs in WWTPs is important to increase the economic feasibility of the treatment system, given that the nitrogen removal process plays a key role in the achievement of energy-neutral WWTPs.

In the case of nitrogen removal processes, N/DN is a nitrogen removal pathway that requires high energy consumption due to the use of electricity for aeration (Maurer et al. 2003). Likewise, the external carbon sources for the denitrification process can lead to operating costs of up to € 3.64/kg N_removed_ (Arias et al. 2018). In a typical WWTP with nitrification-denitrification, the energy consumption can reach 14.6 kWh/kg N_removed_ (Panepinto et al. 2016; Wang et al. 2019; Rodziewicz et al. 2022). In a simulation model, energy consumption during N removal can reach 52 kWh/kg N_removed_ using indicators for loads of total nitrogen removed from dairy wastewater in biological processes (Żyłka et al. 2021).

As for anammox, it shows an improved energy footprint over N/DN. Thus, this technology is considered as the most promising and energy saving nitrogen removal process (Zhang et al. 2021). Evidence demonstrates that it is possible to save energy by up to 44% if the anammox process is implemented in the mainline of a full-scale conventional treatment plant and that it also increases the biogas production yield (+ 9 kWh/per year), which results in an efficient process with net positive energy output (Maktabifard et al. 2018).

Evidence suggests that wastewater itself has more energy than it needs for treatment (Yuan and He 2015). Just ammonium has gained increasing attention as a carbon-free energy source. It is estimated that up to 5.5 kWh per g NH_4_^+^-N is theoretically available from ammonium oxidation (Yüzbaşıoğlu et al. 2022). Thus, harvesting energy from ammonium in WWTPs can contribute to reduce even more the energy demand (Cano et al. 2023).

In fact, the advantages of anammox have driven the development of innovative alternatives that couple this process to other technologies, such as conventional nitrification. In this sense, Jonasson (2007) stated that it is possible to reduce the total electricity consumption of a treatment plant by up to 12% after substituting the N/DN by PNA. This energy saving occurs whenever the PNA demands lower oxygen consumption (−60%) since only approximately half of the ammonium needs to be oxidized to nitrite, equivalent to 0.75 moles of O_2_ per mole of NH_4_^+^oxidized (Soliman and Eldyasti 2018). Similarly, Arias et al. (2018) confirmed the superior energy footprint of the PNA-based technology called ANR. The cost of electrical power of the ELAN process was 0.27 €/m^3^, which represents an energy cost four times lower than that demanded by the conventional N/DN (1.09 €/m^3^). This observation had previously been stated by Lackner et al. (2014). In their case, the power consumption of the N/DN was 4 kWh/kg N_removed_, which represented at least half of the electricity consumption reported for the PNA (0.8–2 kWh/kg N). Nevertheless, energy costs can be even lower if the electrical cost of bioelectrochemical systems is considered. Although, power supplies, recirculation pumps and cathodic aeration pumps are the main energy consumers in BES (Dong et al. 2015), the data show lower energy requirements (see Table 1).

**Table 1:** Power consumption for biological nitrogen removal processes

Process	O_2_ consumption (mole per mole of NH_4_^+^)	Energy consumption (kWh/KgN_removed_)	Reference
N/DN	2	2.7	Schaubroeck et al. (2015)
		2.4	Figueroa et al. (2012)
		2.4–4.3	Joss et al. (2009)
		6	Wett et al. (2015)
		3.5	Vineyard et al. (2020)
		33	Fenu et al. (2019)
PNA	1.5	1.5	Schaubroeck et al. (2015)
		1	Joss et al. (2009)
		1.2	Wett et al. (2015)
		4.2	Lackner et al. (2014)
		1	Figueroa et al. (2012)
Conventional anammox	0.75	1.5	Vineyard et al. (2020)
		1.9	Lackner et al. (2014)
Anammox-MFC	0.75	0.9	Yang et al. (2017)
		1.2	Vilajeliu-Pons et al. (2018)
Anammox-MEC	0.75	6.8	Pous et al. (2015)

From the energy footprint perspective, MFC seem to be the best technology to remove ammonium from wastewater; their energy consumption ranges between 0.9 and 1.2 kWh/kg N. However, energy savings are still on the horizon, since the low consumption of electricity has been obtained with laboratory-scale experiments, so it is still necessary to verify the energy demand that scaling would entail (Xia et al. 2018). Regarding MEC, although there are sufficient studies that apply this technology for nitrogen removal, now there is only one study that states the power expenditure involved in its operation. According to Pous et al. (2015), the energy consumption of a MEC during nitrogen removal is approximately 6.8 kWh/kg N_removed_, which is higher than that required by most technologies applied for nitrogen removal. In this case, the high energy expenditure is explained by the aeration of the cell and by the application of external voltage. Therefore, while the potential for applying BES for nitrogen removal from wastewaters is very promissory, further works should be conducted to optimize and scale up these treatment systems.

## Potential applications of other electron acceptors in nitrogen-rich wastewater

Anammox coupled to the reduction of other electron acceptors, distinct to nitrite, has great potential for ammonium removal from specific wastewaters. These microbial processes represent a novel alternative to replace conventional aerobic and anaerobic ammonium oxidation in wastewaters. As microbe-driven processes, their potential applications in the removal of nitrogen from wastewater must be detailed.

Feammox has great potential advantages for wastewater treatment: (1) It could treat industrial wastewater with high content of heavy metals, as microorganisms involved in this reaction could also reduce additional elements besides Fe (Huang and Jaffé 2015; Weng et al. 2017; Xiu et al. 2020); (2) it does not require aeration, reducing energy consumption and wastewater treatment costs (Liang et al. 2022; Hu et al. 2022); (3) it can be carried out in a wide pH range, especially under acidic conditions; (4) its main product is N_2_, which is harmless to humans and the environment. Furthermore, if NO_2_^−^-N and NO_3_^−^-N are formed, they can be treated by anammox and nitrate-dependent Fe(II) oxidation, respectively (Yang 2020; Zhu et al. 2022; Sun et al. 2023); and finally, 5) the generated Fe(II) can be removed by conventional techniques, such as electrocoagulation and oxidation-precipitation-filtration processes (Zhu et al. 2021).

Sulfammox is also a promising approach for treating nitrogen rich-wastewater, particularly those containing high amounts of sulfurous compounds. This process offers unique advantages for its practical application because: (1) It could be suitable for the simultaneous removal of ammonium and sulfate in different wastewaters, such as those from aquaculture and fish-processing plants, chemical, pharmaceutical, paper mills, sugar production, and textile industries (Yang et al. 2009; Rios-Del Toro and Cervantes 2019); (2) compared to conventional anammox, sulfammox is easier to control as nitritation is not needed (Zhang et al. 2009; Dominika et al. 2021); (3) sulfammox and anammox could coexist and completely remove nitrogen, as the nitrite produced by sulfammox can be used by anammox (Rikmann et al. 2016); (4) since it is a reducing process, the formation of toxic sulfide is avoided (Dominika et al. 2021); (5) it can prevent interference with the conventional anammox caused by inhibition of S^2−^ (Xu et al. 2020), and (6) elemental sulfur has been identified as an intermediate product from Sulfammox process and is therefore conceivable as a valuable by-product (Rios-Del Toro and Cervantes 2019).

Humic substances and graphene oxide have recently been identified as a major opportunity for improving wastewater treatment, because of their enormous potential for developing efficient nitrogen removal techniques. Due to their cost and great reusability, they are advantageous in wastewater treatment, because (1) They exhibit a long-term catalytic effect on the activity of growth cells and activity of anammox bacteria due to their unique structures and properties (Ruiz et al. 2011); (2) their abundant oxygenated functional groups can serve both as electron shuttle and as electron acceptor in extracellular electron transfer among bacteria, which accelerate the anammox activity and increase the nitrogen removal rate (Khadem et al. 2017; Li et al. 2019a); (3) they can remove not only nitrogen but also other pollutants, such as hazardous organic and inorganic chemicals in specific industrial wastewater by adsorption or photodegradation (Kochany and Lipczynska-Kochany 2009); and (4) their application can change EPS concentration, leading to bacterial aggregation (Lu et al. 2021). Additionally, humics and GO can also be immobilized in the anode of BES to enhance the anaerobic ammonium oxidation in the anodic chamber. This is because these electron acceptors share oxygenated functional groups (*e.g*. quinones), which are also present in graphitic anodes.

Alternatively, bioelectrochemical systems have been proposed as a new treatment technology for the removal of ammonium from wastewater in which the anode serves as the electron acceptor. The application of BES for this purpose offers new advantages, such as: (1) ammonium oxidation can release a large number of electrons, so it is considered a potential fuel in bioelectrochemical systems (He et al. 2009); (2) another benefit is that ammonium is oxidized without oxygen, which reduces energy and causes less electron loss (Zhang et al. 2022); (3) anammox bacteria have recently been considered as electrochemically active bacteria that can simultaneously remove nitrogen and produce electricity from wastewater (Shaw et a. 2020); and (4) nitrate and nitrite formed during NH_4_^+^-N oxidation can be reduced to N_2_ at the cathodic chamber (complete denitrification), which increases the efficiency of nitrogen removal (Rodríguez Arredondo et al. 2015).

## Perspectives and future research

Anammox is a cost-effective alternative to conventional nitrogen removal systems. However, the frequent absence of nitrite in wastewater has led the scientific community to search for other electron acceptors, suitable to support the anoxic oxidation of ammonium. From this point of view, new nitrogen removal processes have demonstrated the ability of microorganisms to couple anammox to their respiratory chains with various electron acceptors, including nitric oxide, graphene oxide, sulfate (sulfammox), iron (feammox), natural organic matter (NOM-anammox), and electrodes of BES (anodic-anammox). Although coupling anammox with other electron acceptors has great potential to solve the problem of specific industrial wastewaters with high nitrogen content, the knowledge and understanding of these processes are still limited. Thus, there is still a long way to go before the technologies can be applied in practice. Thus, more research is required to elucidate the potential mechanisms, microbial interactions, and their metabolic pathways that mediate these processes to expand the understanding in terms of the biological nitrogen removal processes and their future application in wastewater treatment systems. These advances will undoubtedly turn the process into an efficient and cost-effective technology.

## Abbreviations

Anammox: anaerobic ammonium oxidation
AQC: anthraquinone-2-carboxylic acid
AQDS: anthraquinone-2,6-disulfonate
ATP: adenosine triphosphate
ANR: autotrophic nitrogen removal
BES: bioelectrochemical systems
BNF: biological nitrogen fixation
EAB: electrochemically active biofilms
EET: extracellular electron transfer
ELAN: Spanish acronym for autotrophic nitrogen elimination
EPS: extracellular polymeric substances
Feammox: anaerobic ammonium oxidation coupled to dissimilatory iron reduction
FISH: fluorescence in-situ hybridization
GO: Graphene oxide
HDH: hydrazine dehydrogenase
HZS: hydrazine synthase
LAW: 2-hydroxy-1-4-naphthoquinone
MEC: microbial electrolysis cells
MFC: microbial fuel cells
NOM: natural organic matter
N/DN: nitrification/denitrification
NiR: nitrite reductase
NR: nitrate reductase
PNA: partial nitritation and anammox
rGO: reduced graphene oxide
SHE: standard hydrogen electrode
sulfammox: sulfate-reducing ammonium oxidation
TN: total nitrogen
WWTPs: wastewater treatment plants
